# Lack of Decorin Expression by Human Bladder Cancer Cells Offers New Tools in the Therapy of Urothelial Malignancies

**DOI:** 10.1371/journal.pone.0076190

**Published:** 2013-10-11

**Authors:** Annele Sainio, Marie Nyman, Riikka Lund, Sanna Vuorikoski, Pia Boström, Matti Laato, Peter J. Boström, Hannu Järveläinen

**Affiliations:** 1 Department of Medical Biochemistry and Genetics, University of Turku, Turku, Finland; 2 Turku Centre for Biotechnology, University of Turku and Åbo Akademi University, Turku, Finland; 3 Department of Pathology, University of Turku, Turku, Finland; 4 Division of Medicine, Department of Endocrinology, Turku University Hospital, Turku, Finland; 5 Division of Digestive Surgery and Urology, Department of Urology, Turku University Hospital, Turku, Finland; INRS, Canada

## Abstract

Decorin, a multifunctional small leucine-rich extracellular matrix proteoglycan, has been shown to possess potent antitumour activity. However, there is some uncertainty whether different cancer cells express decorin in addition to non-malignant stromal cells. In this study we clarified decorin expression by human bladder cancer cells both *in vivo* and *in vitro*. In addition, the effect of adenovirus-mediated decorin expression on human bladder cancer cells *in vitro* was examined. We first demonstrated using the publicly available GeneSapiens databank that decorin gene expression is present in both normal and malignant human bladder tissues. However, when we applied in situ hybridization with digoxigenin-labeled RNA probes for decorin on human bladder carcinoma tissue samples derived from a large radical cystectomy patient cohort (n = 199), we unambiguously demonstrated that invasive and non-invasive bladder carcinoma cells completely lack decorin mRNA. The cancer cells were also negative for decorin immunoreactivity. Instead, decorin expression was localized solely to original non-malignant stromal areas of bladder tissue. In accordance with the aforementioned results, human bladder cancer cells *in vitro* were also negative for decorin expression as shown by RT-qPCR analyses. The lack of decorin expression by bladder cancer cells was shown not to be due to the methylation of the proximal promoter region of the decorin gene. When bladder cancer cells were transfected with a decorin adenoviral vector, their proliferation was significantly decreased. In conclusion, we have shown that human bladder cancer cells are totally devoid of decorin expression. We have also shown that adenovirus-mediated decorin gene transduction of human bladder cancer cell lines markedly inhibits their proliferation. Thus, decorin gene delivery offers new potential therapeutic tools in urothelial malignancies.

## Introduction

Today we understand that extracellular matrix (ECM) macromolecules do not only form an inert space filling microenvironment around the cells, but act as a dynamic structure generating signals to control cell behaviour [1]. Indeed, the ECM and its components including a small leucine-rich proteoglycan decorin [2], [3] are now known to play a central role in a variety of physiological and pathological processes via their capability to regulate key cellular events such as adhesion, migration, proliferation and apoptosis [4].

Small leucine-rich proteoglycans (SLRPs) form a gene family of five subclasses consisting of 18 members, including decorin, the prototype member of the family, and its close relative, biglycan [5]–[6]. Regarding decorin, several splice variants (A1, A2, B–E) have been identified at the mRNA level [7]. Decorin is normally composed of a core glycoprotein with a molecular weight of about 42 kDa and a single chondroitin/dermatan sulfate side chain. In its core glycoprotein there are 10 leucine-rich repeats (LRR), each repeat consisting of 24 amino acids and comprising an α-helix and a β-turn [2], [8]. Decorińs structural features enable it to interact with a number of other ECM proteins, cytokines, growth factors and their receptors such as epidermal growth factor receptor (EGFR), MET (mesenchymal-epithelial transition) receptor, i.e., the receptor for hepatocyte growth factor, insulin-like growth factor receptor I (IGF-IR) and members of ErbB receptor family [8]–[10]. Via these interactions decorin has versatile actions in both health and disease.

The role of decorin in cancer progression and its therapeutic potential as a tumour suppressing antimetastatic agent has been the focus of numerous studies [10]–[11]. Initially, decorin was linked to cancer when it was discovered that decorin/p53 double knockout mice developed tumours faster than controls [10]. The results indicated that disruption of the decorin gene does not lead to spontaneous development of tumours, but lack of decorin is permissive for tumourigenesis [10]. In subsequent studies the expression of decorin has been found to be decreased in several cancers such as colon [12], prostate [13], and ovarian cancers [14]. Furthermore, in breast cancer low expression of decorin has been shown to be associated with a shorter time to progression and a poorer survival [15]. On the other hand, delivery of decorin gene or protein has been demonstrated to lead to growth retardation of different cancers [16]–[18] through various mechanisms of action such as via binding to growth factor receptors mentioned above and modulating their activity [11]. Recently, we have shown that different types of human breast cancer cells totally lack decorin mRNA [19]. We have also shown that the adhesion, proliferation and apoptosis of MCF-7 human breast cancer cells can be influenced by decorin transduction [19].

In bladder cancer, which is the 9^th^ most common cancer diagnosis worldwide [20], the expression of decorin has previously been shown to be decreased [21]–[24]. As decorin acts as a natural antagonist for IGF-IR in tumours, its decreased expression may contribute to increased IGF-IR activity, thus leading to the progression of IGF-IR-dependent cancers through enhanced cellular motility and invasion [23]–[24]. It is also possible that the antitumour effect of decorin on bladder cancer is mediated via decorin-associated tumor suppressor gene mitostatin, whose activity is reduced in advanced bladder cancer alleviating growth and spread of neoplastic cells [25].

Although several studies have examined decorin expression in various cancers including bladder cancer, there is some uncertainty whether different cancer cells express it or not. In this study, we examined the expression of decorin by human bladder cancer cells both *in vivo* and *in vitro*. We also examined the effect of decorin gene transduction on the proliferation of human bladder cancer cells *in vitro*.

## Materials and Methods

### GeneSapiens database

We used GeneSapiens database to evaluate the previously published results regarding decorin expression in normal and malignant human tissues [26]. This database (http://www.genesapiens.org/) covers the relative gene expression patterns for 17 330 genes across all the 9783 annotated normal and pathological human tissue samples from publicly available Affymetrix microarray experiments. In GeneSapiens database there is information on 35 different epithelial carcinomas *in vivo*. For this study, we used decorin expression of 174 samples representing human bladder cancer and compared with data derived from 20 normal human bladder tissue samples.

### Tumour samples

Ethical approval for the use of the clinical material of this study was given by the Ethics Committee of Hospital District of Southwest Finland. The Ethics Committee waived the need for written informed consent. Bladder cancer tissue samples were derived from 199 radical cystectomy patients operated in 1985–2005 in Turku University Hospital, Turku, Finland. The pathological characteristics of the patients are presented in table 1. Tissue microarrays (TMA) were constructed by obtaining 3 representative cores from donor radical cystectomy blocks. We used 5 µm consecutive sections from the TMA blocks for hematoxylin and eosin (HE) staining, in situ hybridization (ISH) and immunohistochemistry (IHC). The use of the samples had the approval of the local ethical committee.

**Table 1:** Clinic pathological characteristics of the study population.

Variable		No.	%
Gender	Male	162	81
	Female	37	19
Age	Years (median, range)	66 (36–80)	
Smoking	Never	88	44
	Former	78	39
	Current	23	12
	Unknown	10	5
Histology	Urothelial	191	96
	Squamous cell carcinoma	5	3
	Adenocarcinoma	3	2
Grade^1^	Grade 1	9	5
	Grade 2	71	36
	Grade 3	119	60
pT-category	≤pT1	94	46
	PT2	41	21
	pT3	50	25
	pT4	14	7
pN-category	N0	58	29
	N1-3	15	8
	Nx^2^	126	63
LVI^3^	Not present	130	65
	Present	69	35
Disease status	Alive with NED^4^	50	25
	Alive with recurrence	1	1
	Death of disease	73	37
	Death of other cause	56	28
	Lost to follow-up	19	10
Follow-up time	Years (median, range)	8.8 (0.2–22.9)	

1 1973 WHO classification, ^2^ Node status unknown, ^3^ Lymphovascular invasion, ^4^ No evident disease.

### Decorin in situ hybridization

We performed decorin ISH on all TMA sections by probing the samples with human decorin antisense and sense single-stranded RNA riboprobes as previously described in detail [27].

### Immunohistochemistry

The IHC analyses were performed for decorin with a rabbit polyclonal antibody (H-80, Santa Cruz Inc., Santa Cruz, CA, dilution 1∶400) and for biglycan with a goat polyclonal antibody (L-15, Santa Cruz Inc., Santa Cruz, CA, dilution 1∶200) on all TMA sections as previously described [27]. Immunostaining for Ki-67 was performed as described below in the section for adenovirus-mediated decorin transduction.

### RT-qPCR for decorin

Three human urinary bladder cancer cell lines RT-4, 5637, and T24 were purchased from American Type Culture Collection (ATCC). These cell lines were used for RT-qPCR analysis for decorin. All the cell lines were maintained in Dulbecco's modified Eagle medium (DMEM; Gibco, Paisley, Scotland) containing 10% fetal bovine serum (FBS; Biochrom AG, Berlin, Germany), penicillin (100 IU/ml) and streptomycin (100 µg/ml) (Sigma, Saint Louis, Missouri, USA), and grown at 37°C with 5% CO_2_. The cells were trypsinized, pooled, and the RNA was extracted using NucleoSpin RNA II –kit (Macherey-Nagel, Düren, Germany) according to the manufacturer's instructions.

RNA concentrations from the cancer cell line extractions were determined using a NanoDrop spectrophotometer (ThermoScientific, Waltham, MA, USA) and the integrity of the RNA was confirmed with agarose gel electrophoresis. One µg of RNA was DNase treated with RQ1 RNase-Free DNase (Promega, Madison, WI, USA) and reverse transcribed into cDNA using M-MLV reverse transcriptase and Oligo(dT)15 primer (Promega, Madison, WI, USA) according to manufacturer's instructions. RT-qPCR was performed as previously described [19].

### DNA methylation status of decorin promoter

Total DNA was extracted from urinary bladder cancer cell lines (RT-4, 5637 and T24) using QIAamp® genomic DNA kit (QIAGEN GmbH, Hilden, Germany) following the manufactureŕs protocol. Methylated DNA was enriched with two different assays, first with the automated MethylCap and then with the automated MeDIP assay by using epigenetic sample preparation robot SX-8G IP-Star® (Diagenode). Briefly, the genomic DNA was first fragmented with Covaris S2 sonicator. The methylated DNA fragments were enriched with Methyl Binding Protein Domain (MBD) affinity based MethylCap assay or 5-methylcytosine antibody based MeDIP immunoprecipitation assay as described by the manufacturer (Diagenode). In order to examine the methylation status of decorin gene promoter, the methylated DNA fragments were purified (MinElute PCR purification kit, Qiagen) and subjected to quantitative PCR (SybrGreenER qPCR Supermix Universal, Invitrogen) by using 7900HT Fast RT-PCR system (Applied Biosystems). Each sample was run in four replicates and three promoter regions covering different decorin isoforms (A1, A2, B–E) were examined. The qPCR oligos used in the RT-PCR analysis were: DCN_A1_F 5′- CAG GTG TGG AAA GGA GGA GG -3′; DCN_A1_R 5′- GTG TCA GCC GGA TTG TGT TC -3′; DCN_A2_F 5′- AGT CCT CAC CTG AAC CCT GA -3′; DCN_A2_R 5′- GAA AGC AGC ATC TTG CCT GG -3′; DCN_B-E _F 5′- CTG CAT CCC ACT CAC CCA AA -3′; DCN_B-E_R 5′- TTC CTG ATG ACC GCG ACT TC -3′. Control loci associated with GAPDH and TSH2B gene promoters (Diagenode) were used as negative and positive controls for DNA methylation, respectively. The recovery % of the methylated DNA was calculated with the formula: recovery % input  = 2? ((Ct input-log input dilution) – CtMeDIP)*100.

### Adenovirus-mediated decorin transduction

For the transduction experiments, a recombinant replication-deficient adenoviral vector dcn-pxc1c-1 was used as previously described [19]. This vector harbors the human decorin (DCN) cDNA under the control of cytomegalovirus (CMV) promoter. For the preparation of the vector, full length human decorin cDNA [28] in pGEM plasmids was cloned and inserted into shuttle plasmid pxcJL-1. The viruses were prepared by cotransfecting HEK293-cells with back bone plasmid pBHG10. As a control vector RAdlacZ, which harbors the E. coli β-galactosidase gene (lacZ) under the control of CMV IE promoter was used. This vector was purchased from the Virus Vector Facility, Centre for Biotechnology, University of Turku, Turku, Finland. Human bladder cancer cell lines RT4 and T24 were used for transduction according to a protocol as previously described [19] with minor modifications. Briefly, cancer cells were maintained in Dulbecco's Modified Eagle Medium (DMEM) containing 10 % fetal bovine serum (FBS), penicillin (100 IU/mL), and streptomycin (100 μg/mL) and grown at 37°C with 5% CO_2_. The cells were plated on an 8-well chamber slide (Thermo Scientific, Rochester, NY, USA), 30 000 cells per well. The next day, the cells were transduced with 10, 100 and 1000 pfu/cell of dcn-pxc1c-1 or RAdlacZ in DMEM containing 10 % FBS. 24 hours after transduction, medium was removed and replaced with fresh one. The cells were then grown until the next day, whereafter they were fixed with 4% paraformaldehyde in phosphate buffered saline (PBS). Finally, the proliferation index of decorin transduced cell lines was determined with a Ki-67 rabbit monoclonal antibody (30–9, Ventana Medical Systems/ Roche Diagnostics, Tucson, Arizona, dilution 1∶200) [19]. Ki-67 positive cells were counted in ten different fields of view (magnification 10×) in decorin and lacZ transfected cell cultures as well as untreated control cultures. Additionally, the number of Ki-67 positive cells/100 cells per field in ten different fields was counted to exclude the possibility that the altered cell number in different cultures would have caused a distortion in the proliferation results. The effect of decorin transduction on cell count was also measured using a haemocytometer. Briefly, the cells were plated on a 12-well plate (Thermo Scientific, Rochester, NY, USA), 170 000 cells per well. Transfection was performed as described above and cells were counted 24 hours after replacing the medium with fresh one. Cell number in each treatment (Ad-DCN, Ad-LacZ Control and Negative Control) was counted as three replicates.

### Statistical analysis

Unpaired Student´s *t* test was employed in statistical analyses. The *p* values <0.05 were considered statistically significant.

## Results

### Relative decorin gene expression in human bladder cancer based on the GeneSapiens in silico transcriptomics data

**Figure 1:**
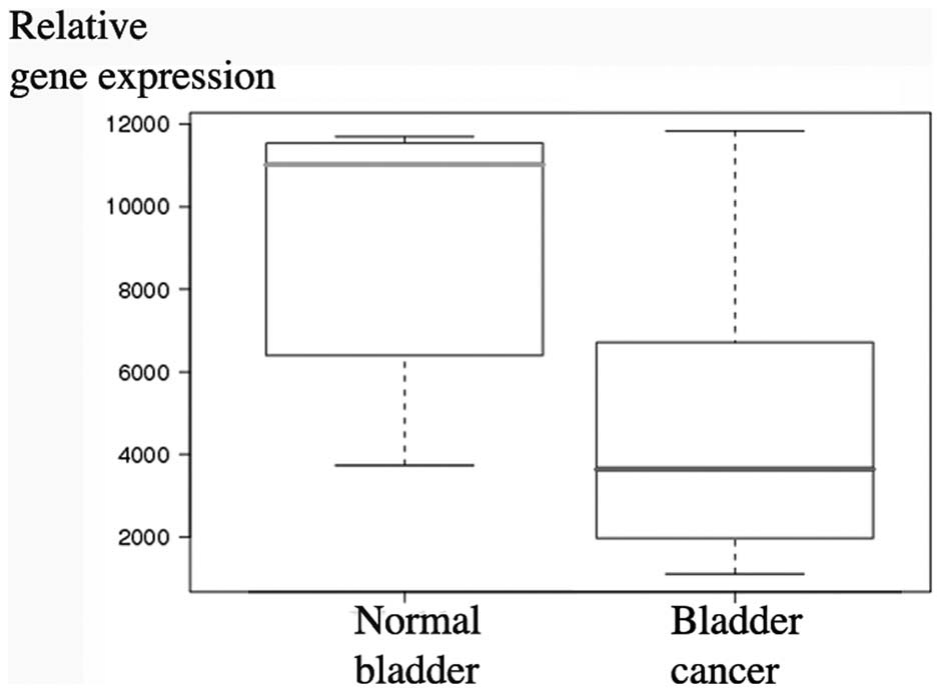
Analysis of decorin expression using GeneSapiens database. Box plot analysis of relative decorin gene expression in tissue samples of normal and malignant human urinary bladder using GeneSapiens *in silico* database (http://www.genesapiens.org/). The continuous lines in the box plot images represent the median expression level of decorin in bladder tissues. Note that relative decorin expression is marked in both normal and malignant bladder tissue samples and that the relative expression of decorin is decreased in bladder cancer compared to normal bladder tissue. Capped bars in the box blot images indicate standard deviations of the results included in the databank.

The GeneSapiens database revealed that decorin is expressed at marked levels in almost all different types of human epithelial carcinoma tissue samples *in vivo* (data not shown) [26]. This was also true for human bladder cancer, although in malignant bladder tissue decorin expression was decreased compared to normal bladder tissue (Figure 1).

### Localization of decorin mRNA and immunoreactivity in malignant human bladder tissue samples

**Figure 2:**
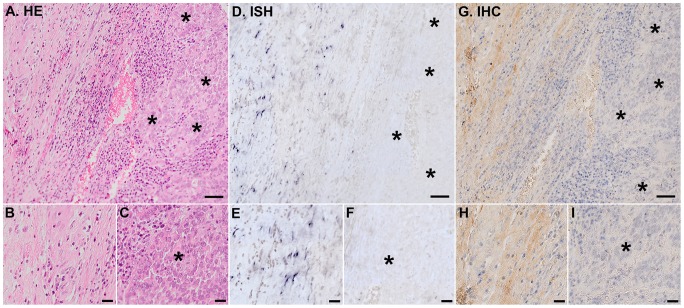
Invasive human bladder cancer cells do not express decorin. Analyses were performed on the whole study population and representative images are shown. Asterisks indicate areas populated solely by bladder cancer cells. Panels A, D, G are representative images of serial sections of the same tissue sample representing invasive human bladder cancer. A. HE staining. D. ISH for decorin. Positive DIG reaction in ISH indicating the localization decorin mRNA can be seen in purple. G. IHC for decorin. Brown color indicates decorin positive nonmalignant stromal cell areas. Parts of normal (B, E, H) and malignant (C, F, I) bladder tissue areas (asterisks) are shown magnified beneath. Note that invasive human bladder carcinoma cells are completely devoid of both decorin mRNA and decorin immunoreactivity and that decorin expression resides solely in the areas of original, non-malignant stromal tissue. In figures A, D, and G, scale bar 50 µm, and in B, C, E, F, H, and I, scale bar 20 µm.

**Figure 3:**
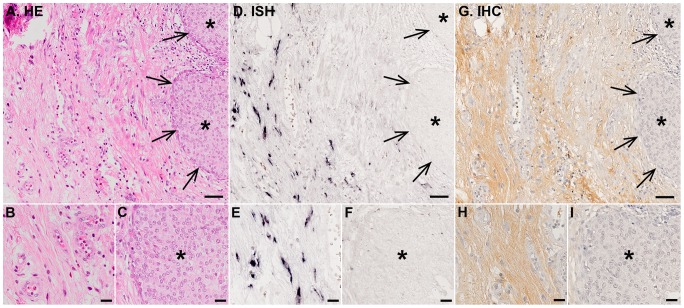
Non-invasive in situ cancer cells do not express decorin. Analyses were performed on the whole study population and representative images are shown. Asterisks indicate areas populated solely by bladder cancer cells. Panels A, D, G are representative images of serial sections of the same tissue sample representing in situ human bladder cancer. Arrows point to the borders of in situ carcinoma and asterisks indicate areas populated by bladder cancer cells only. A. HE staining. D. ISH for decorin. Positive DIG reaction in ISH indicating the localization decorin mRNA can be seen in purple. G. IHC for decorin. Brown color indicates decorin positive nonmalignant stromal cell areas. Parts of normal (B, E, H) and malignant (C, F, I) bladder tissue areas (asterisks) are shown magnified beneath. Note that in situ human bladder carcinoma cells are completely devoid of both decorin mRNA and decorin immunoreactivity and that decorin expression resides solely in the areas of original, non-malignant stromal tissue. In figures A, D, and G, scale bar 50 µm, and in B, C, E, F, H, and I, scale bar 20 µm.

**Figure 4:**
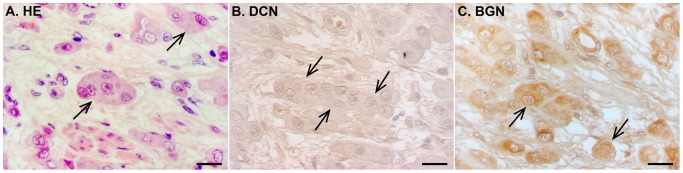
Invasive bladder cancer cells are positive for biglycan immunoreactivity. Arrows indicate examples of malignant bladder cells. A. Representative image of HE staining of invasive bladder cancer tissue. IHC for decorin (B) and biglycan (C) of the same sample as in A. Scale bar in A–C, 20 µm.

The ISH analyses with DIG-labeled RNA probes for decorin clearly demonstrated that invasive bladder carcinoma cells were totally devoid of decorin mRNA in all bladder cancer tissue samples (Figure 2). The same finding was also true for the samples representing non-invasive in situ human bladder cancer (Figure 3). In invasive and in situ bladder carcinomas, all detected decorin mRNA was found to be localized solely to original, non-malignant stromal areas (Figure 2 and 3). The IHC analyses of the samples verified that decorin immunoreactivity resided in the same areas with decorin mRNA (Figure 2 and 3). In contrast, IHC analysis of the samples for another small leucine-rich proteoglycan, namely biglycan, revealed that decorin negative areas in invasive bladder cancer tissue were positive for biglycan immunoreactivity (Figure 4). This finding was true for in situ bladder cancer tissue samples as well (data not shown).

### Decorin expression in human bladder cancer cell lines *in vitro*

**Figure 5:**
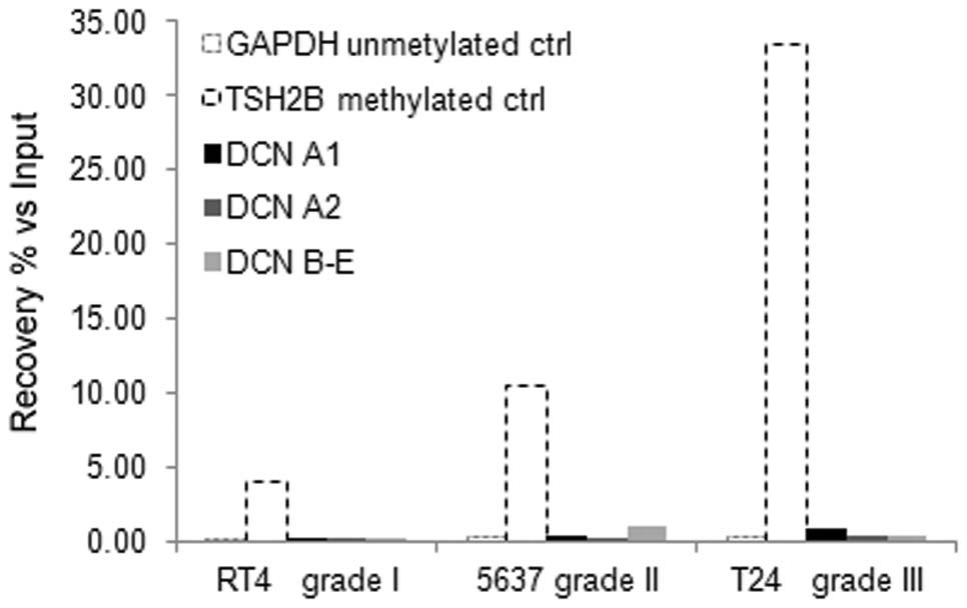
Analysis of the methylation of the decorin gene promoter. Lack of decorin expression in human bladder cancer cell lines is not due to DNA methylation of the decorin gene promoter. Methylation status of decorin isoforms (DCN A1, A2, B–E) in bladder cancer cell lines was studied with two different automated assays, MethylCap and MeDIP. In the figure are quantitative RT-PCR results for MethylCap assay showing % of DNA methylation enrichment versus Input DNA. In addition to decorin gene promoters, the results are shown for positive control TSH2B and negative control GAPDH.

The above *in vivo* results demonstrated that malignant cells within both invasive and non-invasive human bladder cancer tissue samples do not express decorin. Therefore, by using RT-qPCR we next examined whether cell lines representing different grades of human bladder cancer express decorin. The results showed that none of the urinary bladder cancer cell lines, including RT-4 (originally grade I urothelial cancer), 5637 (grade II), and T24 (grade III) expressed decorin. In order to elucidate, whether the lack of decorin expression was due to the DNA methylation of the decorin gene promoter, we used two different assays, MeDIP and MethylCap, followed by quantitative RT-PCR to examine the methylation status of the different decorin gene promoter isoforms extracted from the cancer cell lines. Based on these assays we were not able to detect DNA methylation in the decorin gene promoter in any of the bladder cancer cell lines examined (Figure 5). The control promoter of the TSH2B gene was methylated and GAPDH was not methylated as expected.

### Effect of adenovirus-mediated decorin transduction on the proliferation of human bladder cancer cell lines *in vitro*

**Figure 6:**
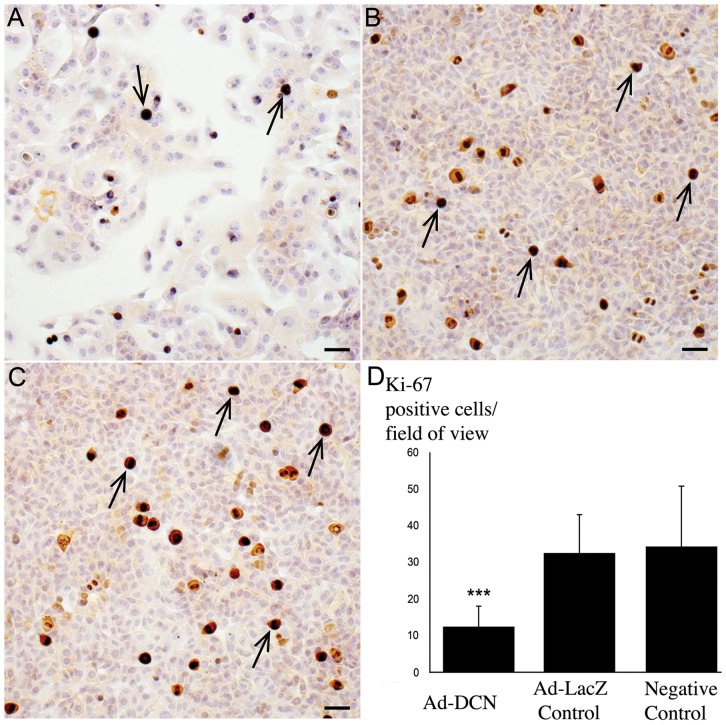
Adenovirus-mediated decorin transduction of human bladder cancer cells. Decorin gene transduction decreases the proliferation index of T24 bladder cancer cells. A. Bladder cancer cells transduced with decorin adenoviral vector (Ad-DCN). B. Transduction of the cells with LacZ vector (Ad-LacZ Control). C. Non-transduced cancer cells (Negative Control). Brown color indicates Ki-67 positive cells (examples indicated by arrows). Scale bar in A-C, 50 µm. Capped bars on top of the columns in D indicate standard deviations. *** P<0.001, Student's *t* test.

Both the ISH results and the RT-qPCR assays clearly demonstrated that human bladder cancer cells are not able to express decorin either *in vivo* or *in vitro*. Next we examined the effect of targeted decorin transduction on the proliferation of human bladder cancer cells *in vitro*. We used human bladder cancer cell lines RT4 and T24 and a decorin adenoviral vector for this purpose. The cells were transduced with a titer of 10–1000 pfu/cell of adenoviral vector and a viral concentration of 1000 pfu/cell was chosen for further experiments. The results showed that adenoviral-mediated decorin transduction decreased the proliferation index of the cancer cells with statistical significance (Figure 6). Also the cell count after decorin transduction was significantly decreased (data not shown).

## Discussion

Although decorińs role in inhibiting tumour growth is recognized today [18], the origin of decorin expression in cancers has remained partially unsolved [12], [13], [23], [29]. Especially, there has been some uncertainty whether different cancer cells express decorin in addition to non-malignant stromal cells. Recently, we have shown that in various forms of human breast cancer, decorin is not expressed by cancer cells [19]. Regarding bladder tissue, the expression of decorin has been shown to be prominent in the subepithelial layers of the murine urinary bladder [30]. It has also been shown with IHC analysis, that decorin immunoreactivity is markedly reduced in the tumour stroma of both low and high grade bladder tumours [23]. In this study, we have examined decorin expression by human bladder cancer cells both *in vivo* and *in vitro*. First, we evaluated the previous data regarding decorin gene expression in different cancers with special reference to bladder cancer utilizing the publicly available GeneSapiens databank [26]. Similarly to previous studies [21]-[22], decorin expression was found to be decreased in malignant human bladder tissue samples compared to normal bladder tissues. Next, we localized decorin mRNA and decorin immunoreactivity in our own extensive radical cystectomy patient cohort of human bladder cancer tissue samples using ISH with DIG-labeled decorin probes and a polyclonal decorin antibody, respectively. As we have shown in human breast cancer [19], these analyses clearly demonstrated that also in human bladder cancer all areas and islets populated by malignant cells were completely devoid of decorin mRNA and immunoreactivity. Instead, the expression of decorin resided solely in the areas of original, non-malignant bladder stroma. Thus, the GeneSapiens results regarding decorin expression in human bladder cancer specimens reflect the quality of the original tissue samples included in the database, i.e., in addition to cancer cells the samples contain various amounts of stromal tissue.

Our *in vivo* results showed that human bladder cancer cells do not express decorin. This same finding was demonstrated to be also true for human bladder cancer cell lines. Because methylation of the decorin gene has previously been shown to regulate decorin expression in colon cancer [29], we decided to examine whether this epigenetic mechanism is affecting decorin expression in human bladder cancer cells as well. However, our results indisputably demonstrated that methylation of decorin gene promoter does not play a role in human bladder cancer. Thus, the mechanisms blocking decorin expression by human bladder cancer cells remain to be elucidated.

Studies utilizing decorin transduction have previously been conducted e.g. with breast cancer cells and the results have shown both reduced primary tumour growth and prevention of metastasis [17], [31]. Furthermore, systemic delivery of decorin protein core to breast carcinoma xenografts has been reported to modulate the expression of several hundred stromal genes creating an unfavourable tumour microenvironment for tumour progression and metastasis [32]. In addition, suppression of tumourigenicity using decorin transduction has also been demonstrated with colon and squamous carcinoma tumour xenografts [16]. Recently, we have shown that adenovirus mediated transduction of decorin to decorin negative breast cancer cells (MCF-7) decreased proliferation and increased apoptosis of the cells [19]. In this study, human bladder cancer cell lines RT4 (gradus I) and T24 (gradus III) were transduced with a decorin adenoviral vector which resulted in an identical decrease in cell proliferation, identical to MCF-7 cells. The aforementioned results together with the observed lack of decorin expression by cancer cells provides an intriguing possibility to examine the effect of decorin on bladder cancer cell behaviour as a therapeutical tool. This could be performed *in vivo* e.g. with intravesical therapy, in which cancerous bladder cavity is rinsed with decorin adenovirus containing fluid [33]–[34].

To the best of our knowledge, the present study is the first to exactly localize decorin mRNA at the cellular level in human bladder cancer *in vivo*. As we screened the samples for the immunoreactivity for another, very similar SLRP to decorin, namely biglycan, we found that the tumour areas negative for decorin were positive for biglycan immunoreactivity. This indicates that although decorin and biglycan represent highly similar molecules that also share similar functions including their ability to bind TGF-β [35] their expression in tissues is not identical. Previously, differential decorin and biglycan expression patters have been found e.g. in developing skeletal and non-skeletal tissues [36], during corneal development [37] and pathological situations such as fibrosis of partial bladder outlet obstruction [38]. Furthermore, biglycan is also gradually proven to act as a key molecule in the regulation of immune system [38]. Based on our results, biglycan expression is detected in human bladder cancer cells which lack decorin expression. The final role of biglycan expression in the progression or restriction of tumourigenesis will require further studies.

## Conclusions

In conclusion, in this study we have shown that human bladder cancer cells do not express decorin either *in vivo* or *in vitro*, and that decorin expression in malignant human bladder tissue resides merely in the areas of original, non-malignant stroma. We have also shown that the lack of decorin expression by human bladder cancer cells is not due to the methylation of the proximal promoter regions of the decorin gene. Furthermore, we have demonstrated that transduction of cultured human bladder cancer cells with a decorin adenoviral vector causes a significant inhibition in the proliferation of the cells *in vitro*. Taken together, our results indicate that the lack of decorin expression by bladder cancer cells offers a possibility for using decorin based therapies in human urothelial malignancies.
